# Life Satisfaction of University Students in Relation to Family and Food in a Developing Country

**DOI:** 10.3389/fpsyg.2017.01522

**Published:** 2017-09-06

**Authors:** Berta Schnettler, Edgardo Miranda-Zapata, Klaus G. Grunert, Germán Lobos, Marianela Denegri, Clementina Hueche, Héctor Poblete

**Affiliations:** ^1^Facultad de Ciencias Agropecuarias y Forestales, Universidad de La Frontera Temuco, Chile; ^2^Centro de Excelencia en Psicología Económica y del Consumo, Núcleo Científico y Tecnológico en Ciencias Sociales, Universidad de La Frontera Temuco, Chile; ^3^LICSA, Núcleo Científico y Tecnológico en Ciencias Sociales, Universidad de La Frontera Temuco, Chile; ^4^MAPP Centre, Aarhus University Aarhus, Denmark; ^5^Facultad de Economía y Negocios, Universidad de Talca Talca, Chile; ^6^Facultad de Educación, Ciencias Sociales y Humanidades, Universidad de La Frontera Temuco, Chile; ^7^Magíster en Sistemas de Gestión Integral de la Calidad, Universidad de La Frontera Temuco, Chile

**Keywords:** family, food, life satisfaction, structural equation modeling, university students

## Abstract

Life satisfaction and satisfaction with food-related life (SWFoL) are associated with healthy eating habits, family interaction around eating and family support. The present study evaluates the relationship between SWFoL and satisfaction with family life (SWFaL), and their relationship with life satisfaction in university students. We identify the relationship of two different types of family support and student SWFaL and explore a moderator effect of gender. A questionnaire was applied to a non-probabilistic sample of 370 students of both genders (mean age 21 years) in Chile, including Satisfaction with Life Scale, SWFoL scale, SWFaL scale, and the Family Resources Scale. Using structural equation modeling, we found that students’ life satisfaction was related to SWFaL and food-related life. A high positive relationship was identified between intangible family support and students’ SWFaL, which would have a mediating role between intangible support and life satisfaction. Using multi-group analysis, a moderator effect of gender was not found. These findings suggest that improving SWFoL, SWFaL and intangible family support is important for both female and male students.

## Introduction

Subjective well-being is a multidimensional category of phenomena involving emotional responses, positive and negative affect, global judgments of life satisfaction and domain satisfactions (Diener et al., 1999). Life satisfaction is thus part of subjective well-being, and bottom–up theoretical approaches to life satisfaction suggest that overall life satisfaction is a combination of satisfaction in definite domains, which implies that life satisfaction depends on the level of satisfaction of a person in regard to life domains such as family, health and leisure, among others (Brief et al., 1993). In addition, different domains often interact (Wilensky, 1960). The spillover model proposes that satisfaction in one domain affects the others and that life domain satisfactions are positively related (Wu et al., 2009). Although the debate regarding the importance of different domains in subjective well-being has endured for decades, the role of domain importance in subjective well-being remains a topic that deserves further research (Hsieh, 2016).

We concentrate in this paper specifically on the role of the domains food and family in university student life satisfaction. While in university, students often face stress personally, socially, academically, economically, or in other areas of life (Çivitci, 2015; Freire et al., 2016). The relationship between family, food, and overall life satisfaction is particularly relevant during undergraduate education, which is evident by the many changes and challenges for emerging adults. During this stage, students often must separate from their family home (Blichfeldt and Gram, 2013). This shift may result in a significant disruption to an individual’s support networks (Beck et al., 2003) as well as economic hardship (Çivitci, 2015) and other personal issues. Therefore, it is expected that although university students seek personal independence, they often continue to rely on their parents for security (Guarnieri et al., 2015). Nevertheless, to our knowledge, no studies have previously assessed the relationship between satisfaction with food-related life (SWFoL), satisfaction with family life (SWFaL), and their joint influence on life satisfaction. Therefore, the primary objective of the current study is to evaluate the joint relationship of these two domains of life, namely family (Zabriskie and Ward, 2013) and food (Grunert et al., 2007), with overall life satisfaction.

Satisfaction with food-related life is defined as a person’s overall assessment regarding his or her food and eating habits (Grunert et al., 2007). Recent studies on samples of undergraduate students suggest that food is an important life domain that is positively related to overall life satisfaction. Schnettler et al. (2013b, 2015a) identified a positive and significant relationship between life satisfaction and SWFoL in samples of university students in southern Chile. Schnettler et al. (2014) explored the relationship between chronic dietary restraint, life satisfaction, and SWFoL, and confirmed the positive and significant correlation between these constructs in a sample of university students from different geographical areas in Chile. In addition, Schnettler et al. (2015c) reported that higher levels of life satisfaction and happiness are associated with greater SWFoL in students from five state universities in Chile. Using a structural equation model, Schnettler et al. (2015b) also found support for a positive and significant correlation between SWFoL and life satisfaction. More recently, Schnettler et al. (2016) confirmed the relationship between these constructs when studying university student perceptions of family eating habits in a sample from southern Chile. Therefore, against this background and based on the “bottom–up” theoretical approach to life satisfaction (Brief et al., 1993), we pose the following hypothesis:

H1: Satisfaction with food-related life is positively related to students’ overall life satisfaction.

Research into family quality of life or SWFaL is a fairly new field (Zabriskie and Ward, 2013; Mas et al., 2016); however, it has been reported that family relationships are strongly linked to subjective well-being (Botha and Booysen, 2014). Higher levels of satisfaction with family relationships are associated with greater adaptability, cohesion and communication (Zabriskie and Ward, 2013), feelings of safety (Rözer et al., 2016), and higher overall life satisfaction (Schimmack et al., 2002). Notably, the influence of family on an individual’s emotional well-being extends beyond childhood and adolescence (Thomson et al., 2015), continuing into emerging adulthood (Tinajero et al., 2015). Positive family relationships and favorable environment conditions may help individuals overcome life challenges, thus enhancing their life satisfaction during youth (Kwok et al., 2015). Schimmack et al. (2002) actually suggest that for university students, family relationships are among the most important sources of life satisfaction. Therefore, again based on the “bottom–up” approach to life satisfaction (Brief et al., 1993), we pose the following hypothesis:

H2: Satisfaction with family life is positively related to students’ overall life satisfaction.

Some authors have related levels of life satisfaction and SWFoL to family interactions involving foods in university students (Schnettler et al., 2013a, 2015a,b, 2016). Food behavior is a learned behavior that reflects a family’s beliefs, attitudes, and practices (Levin and Kirby, 2012). Parents can positively influence their children’s eating habits by providing healthful foods at home and by modeling healthful food choices (Schnettler et al., 2013a, 2015b; Loth et al., 2016; Nepper and Chai, 2016). In fact, access to a healthy food environment at home is associated with a healthy dietary intake (Loth et al., 2016) and with improved family functioning, as it is connected to how family members work together to provide an appropriate home environment and to ensure that each other’s basic needs are met (Speirs et al., 2016). In addition, the relationship between food and family plays an important role in the prevention and treatment of obesity (Levin and Kirby, 2012; Schnettler et al., 2013a; Ramalho et al., 2016), which is also associated with the home food environment (Nepper and Chai, 2016).

The role of food in family relationships also includes the affective dimension of food and meals as a moment of family cohesion (Ramalho et al., 2016; Speirs et al., 2016). Family meals represent an important ritual of interaction among family members, in which these members express their love, maintain close relationships, resolve conflicts, foster cohesion and functioning (Speirs et al., 2016), and give social and emotional support (Schnettler et al., 2015a, 2016; Speirs et al., 2016). Therefore, based on the “spillover” model of the interaction of life domains (Wu et al., 2009), we pose the following hypothesis:

H3: Satisfaction with food-related life is positively related to satisfaction with family life.

Although the literature pertaining to social and family support at different life stages is abundant, there are still few studies on emerging adults, such as university students. Still, it has been reported that higher family social support is related with higher levels of life satisfaction in university students in samples from Spain and the United Kingdom (Goodwin and Hernández, 2000), Mexico and the United States (Matheny et al., 2008; Fakunmoju et al., 2016), China (Kong et al., 2012), Slovenia (Zupančič et al., 2014), and Chile (Schnettler et al., 2015a, 2016). Nevertheless, the relationship between family support and SWFaL has yet to be evaluated in the literature (Zabriskie and Ward, 2013). Considering that there is evidence that family influences the quality of social relationships for adult children (Thomson et al., 2015), it is expected that family support may be positively related with SWFaL. Therefore, the second aim of this study was to assess the relationship between two different types of family support (tangible or financial support and intangible or social support) and student SWFaL. Finally, as a third aim, we chose to explore the possible mediating role of SWFaL between family support and life satisfaction.

Family interaction, emotional well-being, and material or financial well-being are important dimensions that affect family quality of life (Mas et al., 2016). Regarding family interaction and emotional well-being, Rözer et al. (2016) reported that higher levels of satisfaction with family relationships are associated with social support. In addition, social and family support is positively associated with healthy eating habits (Schnettler et al., 2015a; McSpadden et al., 2016). However, the influence of financial well-being on family quality of life may also be considerable (Mas et al., 2016). Cheng et al. (2012) suggest that a high level of family economic support acts as a protective factor, whereas a low level of family economic support becomes a risk factor, thereby lowering life satisfaction and the ability to handle major stressors. However, the evidence is still scarce and inconclusive. Whereas some authors reported a positive relationship between family income or family tangible or economic support and students’ life satisfaction (Schnettler et al., 2015a; Tinajero et al., 2015; Cheung and Cheng, 2016), others have found no relationship between these variables (Menon et al., 2015; Schnettler et al., 2016). Nonetheless, family income or socioeconomic status may impact the nutrition and subjective well-being of emerging adults. Some authors have reported that families from lower socioeconomic backgrounds may not have access to or be able to afford nutritious food for their children (Schnettler et al., 2015b), thereby reducing their children’s life satisfaction and SWFoL (Schnettler et al., 2015b). At the same time, this may affect levels of SWFaL. Against this background, we test the following hypotheses:

H4: Family support received by students, measured in intangible resources, is positively related to students’ satisfaction with family life.
H5: Family support received by students, measured in tangible resources, is positively related to students’ satisfaction with family life.

Feeney and Collins (2015) suggest that social support exists in multiple forms and can affect behavior and well-being through several pathways. Others suggest that certain mediators of the relationship between social support and subjective well-being explain the mechanism underlying this connection (Kong and You, 2013; Liu et al., 2016). In this regard, we explore the mediating role of satisfaction with family life, through the following hypotheses:

H6: Family support received by students, measured in intangible resources, is related to students’ life satisfaction through satisfaction with family life.
H7: Family support received by students, measured in tangible resources, is related to students’ life satisfaction through satisfaction with family life.

Finally, given that males and females differed in some behaviors associated with the constructs evaluated in the model that related life satisfaction, SWFoL, SWFaL, and family support, we expect to find a moderator role of gender. Some authors have reported differences in food choices according to gender. They found that female students tended to make healthier food choices than males (Lupi et al., 2015; Hilger et al., 2017), which have been related to higher levels of SWFoL (Schnettler et al., 2013b, 2014, 2015a,b). In addition, some studies have reported that mealtime experiences may differ for male and female students, with females being more influenced by family relationships. This, in turn, may enable them to benefit more from the shared meal experience (Neumark-Sztainer et al., 2008; Schnettler et al., 2016). At the same time, it has also been reported that female students perceive more support from their families and tend to manifest a better relationship with their parents than male (Tinajero et al., 2015). Meanwhile, some authors have reported that female students are more likely to be satisfied with their life than male students (Ozben, 2013; Tinajero et al., 2015). Against this background, we test the following hypothesis:

H8: Gender moderates the relationships among life satisfaction, satisfaction with food-related life, satisfaction with family life, and family support.

## Materials and Methods

### Sample and Procedure

The students were contacted on campus and asked to participate in a survey. The sample consisted of 370 university students belonging to the six faculties of the University of La Frontera in Temuco, Chile. The inclusion criterion in the sample was enrolment status at the institution at the time of the survey.

Once students agreed to participate, they signed an informed consent prior to completing the survey. A trained surveyor administered the questionnaires during October and December 2015, and the anonymity of the respondents was ensured. A pilot test of the questionnaire was conducted with 35 students from the same university, following the same method of addressing the participants as in the definitive survey. As the pilot test of the instrument was satisfactory, no changes were required in either the questionnaire or the interview procedure. The execution of the study was approved by the Ethics Committee of the Universidad de La Frontera.

The mean age of the sample was 21 years (*SD* = 2.1). Other characteristics of the sample are shown in **Table 1**. The sample obtained presented a similar composition to the population of university students enrolled throughout the country in 2015 in terms of age and gender (Consejo Nacional de Educación [CNED], 2016). The largest proportion live with their parents the entire year or live with their parents only on weekends or for vacations, which is consistent with previous studies with university student samples in Chile (Schnettler et al., 2013a, 2015a,b). Most students belonged to the lower-middle and low socioeconomic status. Education level and occupation of the head of household were used to determine the socioeconomic status (Adimark, 2004).

**Table 1:** Socio-demographic characteristics of the sample, Chile, December 2015 (*n* = 370).

Characteristic	Total
Gender (%)	
Female	54.2
Male	45.8
Age [mean (*SD*)]	21.1 (2.1)
Place of residence during study period (%)	
With parents the entire year	44.6
With parents the entire year although commutes for the day to attend class	15.6
With their parents only on weekends or for vacations	28.1
Independent of parents	11.7
Socioeconomic status (%)	
High and upper-middle	1.5
Middle-middle	16.5
Lower-middle	35.0
Low	38.0
Very low	9.0

### Instrument

The questionnaire included the following scales:

• Satisfaction with Life Scale (SWLS; Diener et al., 1985) is a scale consisting of five items grouped into a single dimension to evaluate overall cognitive judgments about a person’s own life (e.g., “*In most ways my life is close to my ideal*”). The respondents were asked to indicate their degree of agreement with the five statements using a six-level Likert scale (1 = completely disagree to 6 = completely agree). This study used the Spanish-language version of the SWLS which has shown good internal consistency in previous studies with university students in Chile (Schnettler et al., 2013b, 2014, 2015a,b,c, 2016). SWLS score is the sum of the five items of the scale. Higher scores indicate more life satisfaction. The mean SWLS score for all participants was 23.2 (*SD* = 3.96).
• Satisfaction with food-related life (Grunert et al., 2007) is a scale consisting of five items grouped in a single dimension to evaluate a person’s overall assessment regarding their food and eating habits (e.g., “*Food and meals are positive elements*”). Respondents were asked to indicate their degree of agreement with the five statements using a six-level Likert scale (1 = completely disagree to 6 = completely agree). This study used the Spanish-language version of the SWFL which has shown good internal consistency in previous cross-sectional and longitudinal studies with university students in Chile (Schnettler et al., 2013b, 2014, 2015a,b,c, 2016, 2017). The SWFoL score is the sum of the five items of the scale. Higher scores indicate more SWFoL. The mean SWFoL score for all participants was 22.29 (*SD* = 3.94).
• Satisfaction with family life scale, proposed by Zabriskie and McCormick (2003), is a modified version of the SWLS (Diener et al., 1985) in which the words “family life” replaced the word “life” in each of the five original items of the SWLS. Family satisfaction can be defined as a conscious cognitive judgment of one’s family life in which the criteria of the judgment are up to the individual (Zabriskie and Ward, 2013). The SWFaL has shown good internal consistency in previous studies in United States, Canada, United Kingdom, Australia, and New Zealand family samples with their five items grouped in a single dimension (Zabriskie and Ward, 2013). Two bilingual translators translated all original items from English to Spanish. Subsequently, a third bilingual translator back-translated the Spanish version of the scale into English. The differences found were resolved by discussion, with all the translators agreeing on the final versions of the scale. Respondents were asked to indicate their degree of agreement with the five statements using a six-level Likert scale (1 = completely disagree to 6 = completely agree). SWFaL score is the sum of the five items of the scale. Higher scores indicate more SWFaL. The mean SWFaL score for all participants was 23.22 (*SD* = 4.97).
• Family Resources Scale (FRS): developed by Rindfleisch et al. (1997) contains five-point scale items (1 = little or no support to 5 = a lot of support) used to measure the amount of support people receive from their family. Respondents must indicate the amount of total support provided by their family for each of the following categories: Spending money; Food; Clothing; Time and attention; Discipline; Emotional support and love; Life skills and instruction; Role modeling and guidance. The items have been used as two subscales to separately measure intangible or social support (Intan R) and tangible or economic support (Tan R). This study used the Spanish version of the FRS, which has shown good levels of internal consistency in previous studies in Chile for each subscale (Schnettler et al., 2015a, 2016).

### Data Analysis

The two-step procedure recommended by Anderson and Gerbing (1988) was used to measure the relationships between life satisfaction, SWFoL, SWFaL, and family support. First, a measurement model was estimated using a confirmatory factor analysis and a structural equation model was used to test relationships. To conduct confirmatory factor analysis and structural equation model, the software Mplus v. 7.3 was used. Considering the ordinal scale of the items, the polychoric correlation matrix was used to perform the analysis. The polychoric correlation is a coefficient that estimates the linear relationship between two unobserved continuous variables, given only observed ordinal data (Flora and Curran, 2004) such as the Likert scale used in the SWLS, SWFoL, SWFaL, and FRS. The estimation method used was robust unweighted least squares, following Forero et al. (2009).

We estimated four psychometric indices. Considering the ordinal scale of items, Ordinal Alpha (Elosua and Zumbo, 2008) was used to examine the reliability as internal consistency. The average variance extracted (AVE) measured the proportion of variance extracted by a latent factor, compared to the total variance of that factor, including the variances of the measurement error of the factor items. When the AVE is less than 0.50, the variance due to measurement error is greater than the variance due to the construct (Lévy and Varela, 2006). Discriminant validity was obtained by comparing the AVE for each factor with the square of the correlation between scales. Convergent validity was found by inspecting the standardized factor loadings of each scale (ideally > 0.5) as well as their significance (Lévy and Varela, 2006).

Following Lau and Cheung (2012), we tested the mediation role of SWFaL, via a structural equation model through bias-corrected (BC) bootstrap confidence intervals, using 1,000 samples. The mediation role was established when the BC confidence interval for the mediation effect did not contain zero. Factor scores were calculated in order to correlate them and to establish the initial relationship between a pair of constructs. Thus, we were able to identify changes in this relationship and to determine if there was mediation or suppression, according to MacKinnon et al. (2000). Pearson correlation was used when both variables had a normal distribution. When any variable had a distribution different from normal distribution, Spearman rho was used.

The Tuker-Lewis Index (TLI) and the Comparative Fit Index (CFI) were used to determine the model fit to the data. The TLI shows an acceptable fit with a value higher than 0.95, while 0.97 is considered a cutoff to establish a good fit (Schermelleh-Engel et al., 2003). Models with CFI values near 0.95 are considered an acceptable fit (Hu and Bentler, 1999). Besides, root mean square error of approximation (RMSEA) is considered. The RMSEA is a badness of fit measure, thus a good fit is found when its value is lower than 0.06 (Hu and Bentler, 1999). In addition, a good fitting model has a non-significant Chi-square (χ^2^). However, with a large sample, as in the present study, Chi-square can be significant even if the model fits the data. This is why the Chi-square ratio above degrees of freedom or Normed Chi-square (NCS) is also usually interpreted, with values lower than 2 indicating a good fit (Tabachnick and Fidell, 2001).

In addition, we tested the invariance of the measurement model across gender considering configural invariance and measurement invariance (weak invariance, strong invariance and strict invariance) following the procedure proposed by Dimitrov (2010). To determine the achievement of configural invariance, the model Chi-square (χ^2^), NCS, CFI, TLI, and RMSEA were used. However, considering that the Chi-square test is sample size sensitive (MacCallum et al., 2006) and not recommended for comparison of nested models, the Satorra-Bentler scaled Chi-square (Satorra and Bentler, 2001) for nested models difference (SB χ^2^) was used. Thus, to establish that measurement invariance is fulfilled, a non-significant Delta of Satorra-Bentler adjustment of Chi-square was required. Furthermore, the invariance of structural model was evaluated, setting path coefficients and loadings invariant across the groups, to test if there is a moderating role of gender.

## Results

### Reliability and Validity of the Measurement Model

**FIGURE 1:**
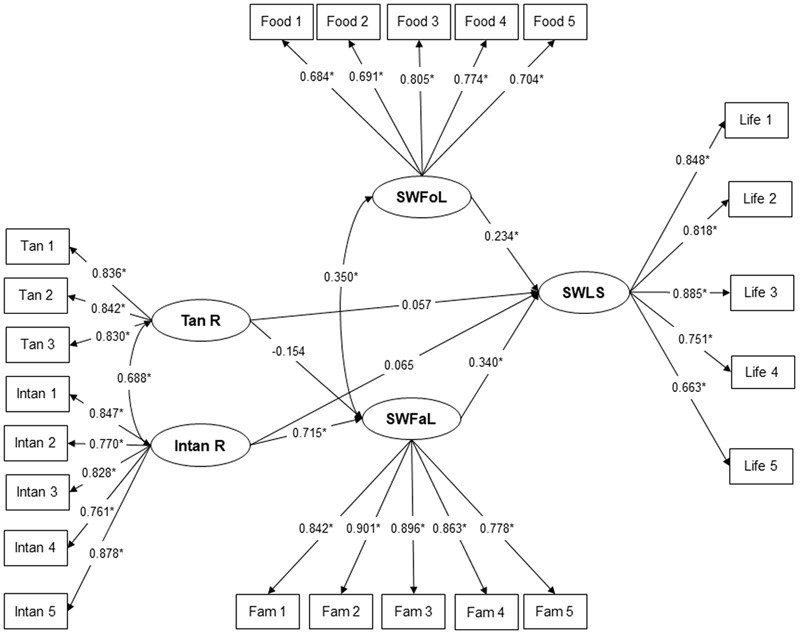
Structural equation model that explains the relationship between satisfaction with food-related life (SWFoL), satisfaction with family life (SWFaL) and student’s Satisfaction with Life (SWLS), and the relationships between Tangible (Tan R) and Intangible (Intan R) resources and student’s SWFaL and Life Satisfaction.^∗^*p* < 0.01. Life 1: In most ways my life is close to my ideal. Life 2: The conditions of my life are excellent. Life 3: 1 am satisfied with my life. Life 4: So far I have gotten the important things I want in life. Life 5: If I could live my life over, I would change almost nothing. Food 1: Food and meals are positive elements. Food 2: I am generally pleased with my food. Food 3: My life in relation to food and meals is close to ideal. Food 4: With regard to food, the conditions of my life are excellent. Food 5: Food and meals give me satisfaction in daily life. Fam 1: In most ways my family life is close to my ideal. Fam 2: The conditions of my family life are excellent. Fam 3: I am satisfied with my family life. Fam 4: So far I have gotten the important things I want in family life. Fam 5: If I could live my family life over, I would change almost nothing. Tan 1: Spending money. Tan 2: Food. Tan 3: Clothing. Intan 1: Time and attention. Intan 2: Discipline. Intan 3: Emotional support and love. Intan 4: Life skills and instruction. Intan 5: Role modeling and guidance.

Results for confirmatory factor analysis indicated that reliabilities of the SWLS, SWFoL, and SWFaL scales and Intan R and Tan R subscales were good (Ordinal alphas above 0.8). The scales and subscales also satisfied the AVE values (above to 0.5) (**Table 2**). The value of the squared correlation between the SWLS and SWFoL was lower than the AVE of the scales, which verified the discriminant validity between the constructs. This finding is consistent with previous studies that sampled adults and university students (Schnettler et al., 2013b, 2015b). The discriminant validity between SWLS and SWFaL, SWFoL and SWFaL, SWLS and Intan R, SWLS and Tan R, SWFoL and Intan R, SWFoL and Tan R, SWFaL and Intan R, SWFaL and Tan R, and between Intan R and Tan R was also verified (Lévy and Varela, 2006). In each scale and subscale, the standardized factor loadings for all items were above 0.5 and statistically significant; thus it was concluded there was convergent validity (**Figure 1**).

**Table 2:** Ordinal alpha, average variance extracted (AVE), correlations and squared correlations between the Satisfaction with Life scale (SWLS), satisfaction with food-related life (SWFoL) scale and satisfaction with family life (SWFaL) scale, intangible resources (Intan R), and tangible resources (Tan R).

Scale/subscale	Ordinal alpha	AVE	SWLS	SWFoL	SWFaL	IntanR	TanR
SWLS	0.89	0.635	–	0.089	0.210	0.074	0.041
SWFoL	0.85	0.528	0.298^∗^	–	0.085	0.008	0.014
SWFaL	0.93	0.735	0.459^∗^	0.292^∗^	–	0.434	0.143
Intan R	0.91	0.669	0.272^∗^	0.091	0.659^∗^	–	0.533
Tan R	0.87	0.698	0.204^∗^	0.118	0.378^∗^	0.730^∗^	–

*^∗^*p* < 0.01. The values over diagonal indicate squared correlations between constructs. The values under diagonal indicate correlations between constructs.*

The model that related life satisfaction, SWFoL, SWFaL and family support was assessed for measurement invariance by gender, starting from the baseline configural invariance followed by factor loadings, thresholds and residuals (**Table 3**). Although the Chi-squared test was significant, the NCS was lower than 2.0. Considering indices of fit, the overall fit of the baseline model is considered good, which established the configural invariance of the scales across gender. This model served as a basis for comparison for the weak or metric measurement invariance model. Then, factor loadings were constrained to be equal across groups to test for weak invariance. The SB χ^2^ difference test between configural invariance (Model 0) and weak invariance (Model 1) models was not significant, demonstrating that the weak invariance was supported. Therefore, factor loadings were invariant across gender. Thus, the items on the scales were related to the latent variables in the same way across gender. Next, equality of thresholds across gender was imposed on the model to test for strong or scalar invariance. The SB χ^2^ difference test between the weak invariance (Model 1) and strong invariance (Model 2) models was not significant, showing that strong invariance was supported. This finding indicates that genders’ mean differences at the observed level reflected the genders’ mean difference at the latent levels. Next, equality of residuals across genders was imposed on the model to test for strict invariance. The SB χ^2^ difference test between the strong invariance (Model 2) and strict invariance (Model 3) models was significant, which means that strict factorial invariance was not supported. This finding indicates that item residuals of the scales were not constant across both student groups.

**Table 3:** Bias-corrected confidence intervals of specific mediation effects.

Effects	Lower 0.5%	Estimate	Upper 0.5%
**From Intan R to SWLS**			
**Specific indirect**			
SWLS			
SWFaL			
Intan R	0.095	0.265	0.477
**From Tan R to SWLS**			
**Specific indirect**			
SWLS			
SWFaL			
Tan R	-0.206	-0.062	0.006

### Testing Hypotheses with Structural Equation Model

Although the structural model (**Figure 1**) had a significant Chi-square value (χ^2^ = 418.392, *p* < 0.01), the NCS (1.88) indicates a good fit. In addition, the rest of the fit indices indicate the structural model had a good fit of the data (CFI = 0.957; TLI = 0.952; RMSEA = 0.049). Path coefficients between SWFoL and SWLS and between SWFaL and SWLS were positive, supporting hypotheses 1 and 2. Nevertheless, according to Cohen (1988), the relationship between SWFoL and SWLS is considered low strength, whereas the relationship between SWFaL and SWLS is considered medium strength. Therefore, the results indicate that a student’s life satisfaction was positively related to their SWFaL, and to a lesser extent, with SWFoL. The positive and significant correlation between the SWFoL and SWFaL indicates that food and family domains interact positively with each other. This finding supports hypothesis 3.

As expected, there was a positive and significant path coefficient between intangible resources and SWFaL, which following Cohen (1988) indicates a high relationship between intangible resources and SWFaL. This finding supports hypothesis 4. Conversely, the negative path coefficient between the tangible resources and the SWFaL was not significant, leading to rejection of hypothesis 5. Considering the change in the value of the correlation between Tan R and SWFaL from positive (rho = 0.378) (**Table 2**) to negative (lambda = -0.154) (**Figure 1**), the presence of a suppression effect is established.

As is presented in **Table 3**, SWFaL has a mediation role between Intan R and SWLS, since the 99% BC confidence interval does not contain zero (lower 0.5% limit = 0.095; upper 0.5% limit = 0.477), indicating that the mediation role is significantly different from zero. This result supports hypothesis 6. This mediation is complete, as there was a statistically significant relation in the absence of mediators, but the relation has been reduced to zero in the presence of the mediator (**Table 4**), given the inclusion of zero in the 99% confidence interval for the relationship between Intan R and SWLS (lower 0.5% limit = -0.357; upper 0.5% limit = 0.275). Otherwise, SWFaL has no mediation role between Tan R and SWLS, since the 99% BC confidence interval does contain zero (lower 0.5% limit = -0.206; upper 0.5% limit = 0.006). This result does not support hypothesis 7. So, the relationships of Intan R and Tan R with SWLS are reduced to zero in the presence of mediators (**Table 4**), but the relationship is only statistically significant for Intan R and SWLS throug SWFaL.

**Table 4:** Confidence intervals of direct effects of Intan R and Tan R in the mediation model.

Effects	Lower 0.5%	Estimate	Upper 0.5%
**SWLS on**			
Intan R	-0.357	-0.065	0.275
Tan R	-0.186	0.058	0.296
**SWFaL on**			
Intan R	0.527	0.722	0.934
Tan R	-0.453	-0.169	0.044

In order to test the moderator role of gender, we used the strong invariance fulfilled as a basis and added the structural path coefficient invariance (Strong and paths, **Table 5**). We found good fit indices and the achievement of strong and paths invariance by gender, which allows us to establish that gender is not a moderator role in the structural model relationships. Therefore, hypothesis 8 must be rejected.

**Table 5:** Factorial Invariance for the model that related life satisfaction, satisfaction with food-related life, satisfaction with family life, and family support between gender.

Model	χ^2^	NCS	D_SB χ^2^	p_D_SB χ^2^	RMSEA	CFI	TLI
0 Configural (without invariance)	677.378	1.46	–	–	0.050	0.950	0.945
1 Metric (loadings invariance)	672.220	1.39	17.313	0.502	0.046	0.955	0.953
2 Strong (loadings and thresholds)	731.262	1.33	80.705	0.158	0.042	0.958	0.961
3 Strict (loadings, thresholds, and residuals)	761.214	1.33	42.024	0.009	0.042	0.956	0.961
4 Strong and path	721.407	1.30	5.312	0.976	0.040	0.961	0.964

*χ^*2*^, Chi square; NCS, Normed Chi square; SB χ^*2*^, Satorra-Bentler adjustment of Chi-square; D, delta between a model and previous model; D_SB χ^*2*^, delta of Satorra-Bentler adjustment of Chi-square; p_D_SB χ^*2*^, statistical significance of delta of Satorra-Bentler adjustment of Chi-square; RMSEA, root mean square error of approximations; CFI, Comparative Fit Index; TLI, Tuker-Lewis Index.*

## Discussion

The primary focus of this study was to assess the relation between SWFoL and SWFaL, and their relationships with life satisfaction in university students. Results of the confirmatory factor analysis confirm the positive relationship between SWFoL and life satisfaction in university students (Schnettler et al., 2013b, 2014, 2015a,c, 2016). According to the “bottom–up” theoretical approach to life satisfaction (Brief et al., 1993), results of the structural equation model analysis also confirm the positive relation between SWFoL and overall life satisfaction reported previously in a similar sample (Schnettler et al., 2015b). These findings confirm that food is a domain that could have an impact on the life satisfaction of a student within a period of life marked by the difficulty of achieving a balanced diet and associated health problems (Blichfeldt and Gram, 2013; Çivitci, 2015; Hilger et al., 2017). Also, the confirmatory factor analysis and structural equation model analysis results suggest a positive relationship between SWFaL and overall life satisfaction in university students, which also is in agreement with the “bottom–up” approach to life satisfaction (Brief et al., 1993) and confirms the importance of family relationships as a source of life satisfaction in university students (Schimmack et al., 2002). It is noteworthy that the relation between SWFoL and life satisfaction was smaller than the relation between SWFaL and life satisfaction. Pavot and Diener (1993) argued that although there may be some general agreement on the components of high well-being, individuals are likely to assign different weights to each component (Zabriskie and Ward, 2013). Therefore, our results indicate that the family domain would be more important than the food domain for the university students sample considered in this study, although university students become increasingly involved in other contexts beyond their family, such as peers, romantic relationships, work and higher education (Guarnieri et al., 2015). Regardless of the above, results of the confirmatory factor analysis and structural equation model show that there is a positive interaction between the domains food and family in the studied sample, consistent with the “spillover” model (Wu et al., 2009). This finding is in line with previous studies using samples of university students that have linked SWFoL to family interaction around foods (Schnettler et al., 2013b, 2015a,b, 2016), which would be associated with the affective dimension of food and meals (Ramalho et al., 2016; Speirs et al., 2016) that increase the well-being of family members given its positive association with social skills and self-esteem (Levin and Kirby, 2012).

The relationship between family and food has also been associated with the moment when family members offer each other support, especially when parents emotionally support their children (Schnettler et al., 2015a, 2016; Speirs et al., 2016). Regarding our second objective, as expected, the structural equation model analysis indicates a positive relation between intangible or emotional resources and student SWFaL, in agreement with Rözer et al. (2016). However, it is noteworthy that the relation was strong, suggesting that intangible family support is of great importance for students during the university phase, increasing their level of SWFaL. In fact, family social support has been shown to be one of the most important protective factors for emerging adults (Tinajero et al., 2015). Regarding our third aim, our results show that SWFaL has a complete mediation role between intangible resources and overall life satisfaction, indicating a non-significant relationship between intangible support and students’ life satisfaction. In this regard, although some researchers have previously reported a positive correlation between family social support and life satisfaction (Goodwin and Hernández, 2000; Matheny et al., 2008; Kong et al., 2012; Zupančič et al., 2014; Schnettler et al., 2015a, 2016; Fakunmoju et al., 2016), our findings are in line with research reporting the existence of mediators in the relation between social support and subjective well-being that explain the mechanism underlying this link, as loneliness and self-esteem (Kong and You, 2013) and core self-evaluations and coping styles (Liu et al., 2016). Therefore, both intangible family support and SWFaL may still have a central role in the development of a university student.

Conversely, there was no association between tangible resources and SWFaL, which indicates that SWFaL does not have a mediating role between tangible support from family and life satisfaction in the studied sample. At the same time, our results show no association between tangible resources and student’s life satisfaction. These were unexpected results that deserves more analysis in future research, given that university is a time when students may be responsible for their economic situation and economic hardship may be a source of stress for students (Çivitci, 2015). Although more research is needed in order to deeply study when tangible or economic resources could have a positive relation with SWFaL and with life satisfaction, a possible explanation may be related to the socioeconomic status composition of the sample. More than 80% of the students surveyed belonged to the lower-middle, low and very low socioeconomic status, therefore it is expected that tangible or economic resources may be insufficient in most of these households. Another unexpected finding was the lack of a moderator role of gender in the model that relates life satisfaction, SWFoL, SWFaL and family resources. Therefore, interventions that promote and strengthen these aspects may positively impact the life satisfaction of a student, regardless of their gender.

One of the limitations of this study include its cross-sectional design and the use of a survey to obtain the data, which does not allow us to test causality among SWFoL and life satisfaction, and between SWFaL and students life satisfaction, as well as between intangible resources and SWFaL. Therefore, in order to test causality between the aforementioned constructs, new research is required that considers experimental or quasi-experimental designs. Another limitation is related to the non-probabilistic nature of the sample and its relatively small size, as well as having been conducted with students from only one university in one country, which does not permit generalization of the results. Also, all data were self-reported, thus, responses may be affected by social desirability.

In spite of these limitations, this is the first study to assess the relation between SWFoL and SWFaL and their relationship with life satisfaction, suggesting both life domains have a positive relationship with the life satisfaction of university students, whereas both domains interact positively with each other. These findings allow the suggestion that interventions to improve the levels of SWFoL and SWFaL may improve life satisfaction of university students in developing countries in South America. In addition, this is the first study to test the relationship between two different types of family support and student SWFaL. Our findings showed a high positive relationship between intangible or emotional family support and SWFaL. In addition, we found that SWFaL may be another mediating factor between intangible family support and life satisfaction, contributing to the knowledge about mediators of the relation between social support and subjective well-being. Therefore, both intangible family support and SWFaL should be promoted in order to improve life satisfaction of university students, who are in a stage of life characterized by many challenges, stress for different reasons, nutritional vulnerability and the possibility of facing health-related problems, both physically and mentally, that will negatively impact their future quality of life. In addition, our findings show that improving SWFoL, SWFaL and intangible family support is important for both female and male students.

## Ethics Statement

This study was conducted in accordance with the recommendations of the Ethics Committee of the Universidad de La Frontera, with the written informed consent of all subjects. All subjects gave written informed consent in accordance with the Declaration of Helsinki. The protocol was approved by Ethics Committee of the Universidad de La Frontera.

## Author Contributions

BS idealized and wrote the first manuscript draft and approved the statistical analysis. EM-Z guided the statistical analysis and made a critical analysis of the final version of the manuscript. GL, MD, and HP supervised data collection and made a critical analysis of the final version of the manuscript. KG made a critical analysis of the final version of the manuscript. CH prepared the literature review. All authors read and approved the final manuscript.

## Conflict of Interest Statement

The authors declare that the research was conducted in the absence of any commercial or financial relationships that could be construed as a potential conflict of interest.

## References

[B1] Adimark (2004). *Mapa Socioeconómico de Chile.* Available at: http://www.adimark.cl

[B2] Anderson J. C. Gerbing D. W. (1988). Structural equation modeling in practice: a review and recommended two-step approach. *Psychol. Bull.* 103 411–423. 10.1037/0033-2909.103.3.411

[B3] Beck R. Taylor C. Robbins M. (2003). Missing home: sociotropy and autonomy and their relationship to psychological distress and homesickness in college freshmen. *Anxiety Stress Coping* 16 155–166. 10.1080/1061580021000056979

[B4] Blichfeldt B. S. Gram M. (2013). Lost in Transition? Student food consumption. *High. Educ.* 65 277–289. 10.1007/s11205-013-0351-6

[B5] Botha F. Booysen F. (2014). Family functioning and life satisfaction and happiness in South African Households. *Soc. Indic. Res.* 119 163–182. 10.1007/s11205-013-0485-6

[B6] Brief A. P. Butcher A. H. George J. M. Link K. E. (1993). Integrating bottom-up and top-down theories of subjective well-being: the case of health. *J. Pers. Soc. Psychol.* 64 646–653. 10.1037/0022-3514.64.4.646 8473981

[B7] Cheng W. Ickes W. Verhofstadt L. (2012). How is family support related to students’ GPA scores? A longitudinal study. *High. Educ.* 64 399–420. 10.1007/s10734-011-9501-4

[B8] Cheung C. K. Cheng J. Y. S. (2016). Resources and norms as conditions for well-being in Hong Kong. *Soc. Indic. Res.* 126 757–775. 10.1007/s11205-015-0901-1

[B9] Çivitci A. (2015). The moderating role of positive and negative affect on the relationship between perceived social support and stress in college students. *Educ. Sci. Theory Pract.* 15 565–573. 10.12738/estp.2015.3.2553

[B10] Cohen J. (1988). *Statistical Power Analysis for the Behavioral Sciences.* Hillsdale, NJ: Erlbaum.

[B11] Consejo Nacional de Educación [CNED] (2016). *Estadísticas y Bases de datos INDICES.* Available at: http://www.cned.cl/public/Secciones/SeccionIndicesEstadisticas/indices_estadisticas.aspx

[B12] Diener E. Emmons R. Larsen R. Griffin S. (1985). The satisfaction with life scale. *J. Pers. Assess.* 49 71–75. 10.1207/s15327752jpa4901_13 16367493

[B13] Diener E. Suh E. Lucas R. Smith H. (1999). Subjective well-being: three decades of progress. *Psychol. Bull.* 125 276–302. 10.1037/0033-2909.125.2.276

[B14] Dimitrov D. M. (2010). Testing for factorial invariance in the context of construct validation. *Meas. Eval. Couns. Dev.* 43 121–149. 10.1177/0748175610373459

[B15] Elosua P. Zumbo B. D. (2008). Coeficientes de fiabilidad para escalas de respuesta categórica ordenada. *Psicothema* 20 896–901. 18940100

[B16] Fakunmoju S. Donahue G. R. McCoy S. Mengel A. S. (2016). Life satisfaction and perceived meaningfulness of learning experience among first-year traditional graduate social work students. *J. Educ. Pract.* 7 49–62.

[B17] Feeney B. C. Collins N. L. (2015). A new look at social support: a theoretical perspective on thriving through relationships. *Pers. Soc. Psychol. Rev.* 19 113–147. 10.1177/1088868314544222 25125368 PMC5480897

[B18] Flora D. B. Curran P. J. (2004). An empirical evaluation of alternative methods of estimation for confirmatory factor analysis with ordinal data. *Psychol. Methods* 9 466–491. 10.1037/1082-989X.9.4.466 15598100 PMC3153362

[B19] Forero C. G. Maydeu-Olivares A. Gallardo-Pujol D. (2009). Factor analysis with ordinal indicators: A Monte Carlo study comparing DWLS and ULS estimation. *Struct. Equ. Model. A Multidiscip. J.* 16 625–641. 10.1080/10705510903203573

[B20] Freire C. Ferradás M. M. Valle A. Núñez J. C. Vallejo G. (2016). Profiles of psychological well-being and coping strategies among university students. *Front. Psychol.* 13:1554 10.3389/fpsyg.2016.01554 PMC5062019 27790168

[B21] Goodwin R. Hernández S. (2000). Perceived and received social support in two cultures: collectivism and support among British and Spanish students. *J. Soc. Pers. Relationsh.* 17 282–291. 10.1177/0265407500172007

[B22] Grunert K. G. Dean M. Raats M. M. Nielsen N. A. Lumbers M. (2007). A measure of satisfaction with food-related life. *Appetite* 49 486–493. 10.1016/j.appet.2007.03.010 17481776

[B23] Guarnieri S. Smorti M. Tani F. (2015). Attachment relationships and life satisfaction during emerging adulthood. *Soc. Indic. Res.* 121 833–847. 10.1007/s11205-014-0655-1

[B24] Hilger J. Loerbroks A. Diehl K. (2017). Eating behaviour of university students in Germany: dietary intake, barriers to healthy eating and changes in eating behaviour since the time of matriculation. *Appetite* 109 100–107. 10.1016/j.appet.2016.11.016 27864073

[B25] Hsieh C. M. (2016). Domain importance in subjective well-being measures. *Soc. Indic. Res.* 127 777–792. 10.1007/s11205-015-0977-7

[B26] Hu L. Bentler P. M. (1999). Cutoff criteria for fit indexes in covariance structure analysis: conventional criteria versus new alternatives. *Struct. Equ. Model. A Multidiscip. J.* 6 1–55. 10.1080/10705519909540118

[B27] Kong F. You X. (2013). Loneliness and self-esteem as mediators between social support and life satisfaction in late adolescence. *Soc. Indic. Res.* 110 271–279. 10.1007/s11205-011-9930-6

[B28] Kong F. Zhao J. You X. (2012). Emotional intelligence and life satisfaction in Chinese university students: the mediating role of self-esteem and social support. *Pers. Individ. Dif.* 53 1039–1043. 10.1016/j.paid.2012.07.032

[B29] Kwok S. Cheng L. Wong D. (2015). Family emotional support, positive psychological capital and job satisfaction among Chinese white-collar workers. *J. Happ. Stud.* 16 561–582. 10.1007/s10902-014-9522-7

[B30] Lau R. S. Cheung G. W. (2012). Estimating and comparing specific mediation effects in complex latent variable models. *Org. Res. Methods* 15 3–16. 10.1177/1094428110391673

[B31] Levin K. A. Kirby J. (2012). Irregular breakfast consumption in adolescence and the family environment: underlying causes by family structure. *Appetite* 59 63–70. 10.1016/j.appet.2012.03.016 22446725

[B32] Lévy J. P. Varela J. (2006). *Modelización con Estructuras de Covarianzas en Ciencias Sociales: Temas Esenciales, Avanzados y Aportaciones Especiales.* Madrid: Netbiblo 544.

[B33] Liu W. Li Z. Ling Y. Cai T. (2016). Core self-evaluations and coping styles as mediators between social support and well-being. *Pers. Individ. Dif.* 88 35–39. 10.1016/j.paid.2015.08.044

[B34] Loth K. A. MacLehose R. F. Larson N. Berge J. M. Neumark-Sztainer D. (2016). Food availability, modeling and restriction: How are these different aspects of the family eating environment related to adolescent dietary intake? *Appetite* 96 80–86. 10.1016/j.appet.2015.08.026 26327222 PMC4684786

[B35] Lupi S. Bagordo F. Stefanati A. Grassi T. Piccinni L. Bergamini M. (2015). Assessment of lifestyle and eating habits among undergraduate students in northern Italy. *Annali dell’Istituto Superiore Sanità* 51 154–161. 10.4415/ANN_15_02_14 26156187

[B36] MacCallum R. C. Browne M. W. Cai L. (2006). Testing differences between nested covariance structure models: power analysis and null hypotheses. *Psychol. Methods* 11 19–35. 10.1037/1082-989X.11.1.19 16594765

[B37] MacKinnon D. P. Krull J. L. Lockwood C. M. (2000). Equivalence of the mediation, confounding and suppression effect. *Prev. Sci.* 1 173 10.1023/A:1026595011371 PMC2819361 11523746

[B38] Mas J. M. Baqués N. Balcells-Balcells A. Dalmau M. Giné C. Gràcia M. (2016). Family quality of life for families in early intervention in Spain. *J. Early Interv.* 38 59–74. 10.1177/1053815116636885

[B39] Matheny K. Roque-Tovar B. Curlette W. (2008). Perceived stress, coping resources, and life satisfaction among U.S. and Mexican college students: a cross-cultural study. *An. Psicol.* 24 49–57.

[B40] McSpadden K. E. Patrick H. Oh A. Y. Yaroch A. L. Dwyer L. A. Nebeling L. C. (2016). The association between motivation and fruit and vegetable intake: the moderating role of social support. *Appetite* 96 87–94. 10.1016/j.appet.2015.08.031 26321416 PMC4684708

[B41] Menon M. Pendakur R. Perali F. (2015). All in the family: how do social capital and material wellbeing affect relational wellbeing? *Soc. Indic. Res.* 124 899–910. 10.1007/s11205-014-0816-2

[B42] Nepper M. J. Chai W. (2016). Parents’ barriers and strategies to promote healthy eating among school-age children. *Appetite* 103 157–164. 10.1016/j.appet.2016.04.012 27090341

[B43] Neumark-Sztainer D. Eisenberg M. E. Fulkerson J. A. Story M. Larson N. I. (2008). Family meals and disordered eating in adolescents: longitudinal findings from project EAT. *Arch. Pediatr. Adolesc. Med.* 162 17–22. 10.1001/archpediatrics.2007.9 18180407

[B44] Ozben S. (2013). Social skills, life satisfaction, and loneliness in Turkish university students. *Soc. Behav. Pers. Int. J.* 41 203–214. 10.2224/sbp.2013.41.2.203

[B45] Pavot W. Diener E. (1993). Review of the satisfaction with life scale. *Soc. Indic. Res.* 39 101–117. 10.1037/1040-3590.5.2.164

[B46] Ramalho J. D. A. M. Lachal J. Bucher-Maluschke J. S. Moro M. R. Revah-Levy A. (2016). A qualitative study of the role of food in family relationships: an insight into the families of Brazilian obese adolescents using photo elicitation. *Appetite* 96 539–545. 10.1016/j.appet.2015.10.023 26505289

[B47] Rindfleisch A. Burroughs J. E. Denton F. (1997). Family structure, materialism, and compulsive consumption. *J. Consum. Res.* 23 312–325.

[B48] Rözer J. Mollenhorst G. Poortman A. R. (2016). Family and friends: Which types of personal relationships go together in a network? *Soc. Indic. Res.* 127 809–826. 10.1007/s11205-015-0987-5 27239093 PMC4863920

[B49] Satorra A. Bentler P. M. (2001). A scaled difference chi-square test statistic for moment structure analysis. *Psychometrika* 66 507–514. 10.1007/BF02296192 PMC2905175 20640194

[B50] Schermelleh-Engel K. Moosbrugger H. Müller H. (2003). Evaluating the fit of structural equation models: tests of significance and descriptive goodness-of-fit measures. *Methods Psychol. Res. Online* 8 23–74.

[B51] Schimmack U. Diener E. Oishi S. (2002). Life-satisfaction is a momentary judgment and a stable personality characteristic: the use of chronically accessible and stable sources. *J. Pers.* 70 345–384. 10.1007/s11205-015-0886-9 12049164

[B52] Schnettler B. Denegri M. Miranda H. Sepúlveda J. Orellana L. Paiva G. (2013a). Hábitos alimentarios y bienestar subjetivo en estudiantes universitarios del sur de Chile. *Nutr. Hosp.* 28 2221–2228. 10.3305/nutr 24506404

[B53] Schnettler B. Denegri M. Miranda H. Sepúlveda J. Orellana L. Paiva G. (2015a). Family support and subjective well-being: an exploratory study of university students in southern Chile. *Soc. Indic. Res.* 1223 833–864. 10.1108/00070700210418767

[B54] Schnettler B. Höger Y. Orellana L. Sepúlveda J. Salinas-Oñate N. Lobos G. (2016). Family eating habits, family support and subjective well-being in university students in Chile. *Nutr. Hosp.* 33 451–458. 10.1016/j.appet.2015.02.008

[B55] Schnettler B. Miranda H. Lobos G. Orellana L. Sepúlveda J. Denegri M. (2015b). Eating habits and subjective well-being: a typology of students in Chilean state universities. *Appetite* 89 203–214. 10.1016/j.appet.2015.02.008 25675858

[B56] Schnettler B. Miranda H. Miranda-Zapata E. Salinas-Oñate N. Grunert K. G. Lobos G. (2017). Longitudinal multigroup invariance analysis of the satisfaction with food-related life scale in university students. *Appetite* 113 91–99. 10.1016/j.appet.2017.02.020 28215544

[B57] Schnettler B. Miranda H. Sepúlveda J. Denegri M. Mora M. Lobos G. (2013b). Psychometric properties of the Satisfaction with Food-Related Life Scale: application in southern Chile. *J. Nutr. Educ. Behav.* 45 443–449. 10.1016/j.jneb.2012.08.003 23337474

[B58] Schnettler B. Miranda H. Sepúlveda J. Orellana L. Etchebarne S. Lobos G. (2014). Dietary restraint and subjective well-being in university students in Chile. *Nutr. Hosp.* 30 453–461. 10.3305/nh.2014.30.2.7561 25208802

[B59] Schnettler B. Orellana L. Lobos G. Miranda H. Sepúlveda J. Etchebarne S. (2015c). Relationship between the domains of the Multidimensional Students’ Life Satisfaction Scale, satisfaction with food-related life and happiness in university students. *Nutr. Hosp.* 31 2752–2763. 10.3305/nh.2015.31.6.9279 26040392

[B60] Speirs K. E. Hayes J. T. Musaad S. VanBrackle A. Sigman-Grant M. All T. (2016). Is family sense of coherence a protective factor against the obesogenic environment? *Appetite* 99 268–276. 10.1016/j.appet.2016.01.025 26796029

[B61] Tabachnick B. Fidell L. (2001). *Using Multivariate Statistics.* New York, NY: Harper & Row.

[B62] Thomson K. Schonert-Reichl K. Oberle E. (2015). Optimism in early adolescence: relations to individual characteristics and ecological assets in families, schools, and neighborhoods. *J. Happ. Stud.* 16 889–913. 10.1007/s10902-014-9539-y

[B63] Tinajero C. Martínez-López Z. Rodríguez M. S. Guisande M. A. Páramo M. F. (2015). Gender and socioeconomic status differences in university students’ perception of social support. *Eur. J. Psychol. Educ.* 30 227–244. 10.1007/s10212-014-0234-5

[B64] Wilensky H. L. (1960). Work, careers, and social integration. *Int. Soc. Sci. J.* 4 543–560.

[B65] Wu C. H. Chen L. H. Tsai Y. M. (2009). Longitudinal invariance analysis of the satisfaction with life scale. *Pers. Individ. Dif.* 46 396–401. 10.1016/j.paid.2008.11.002

[B66] Zabriskie R. McCormick B. (2003). Parent and child perspectives of family leisure involvement and satisfaction with family life. *J. Leis. Res.* 35 163–189.

[B67] Zabriskie R. B. Ward P. J. (2013). Satisfaction with family life scale. *Marriage Fam. Rev.* 49 446–463. 10.1080/01494929.2013.768321

[B68] Zupančič M. Komidar L. Levpušček M. P. (2014). Individuation in Slovene emerging adults: its associations with demographics, transitional markers, achieved criteria for adulthood, and life satisfaction. *J. Adolesc.* 37 421–433. 10.1016/j.adolescence.2014.03.014 24767635

